# The Yin and Yang of Myeloid Derived Suppressor Cells

**DOI:** 10.3389/fimmu.2018.02776

**Published:** 2018-11-28

**Authors:** Snehil Budhwar, Priyanka Verma, Rachna Verma, Sangeeta Rai, Kiran Singh

**Affiliations:** ^1^Department of Molecular and Human Genetics, Institute of Science, Banaras Hindu University, Varanasi, India; ^2^Department of Obstetrics and Gynecology, Institute of Medical Sciences, Banaras Hindu University, Varanasi, India

**Keywords:** myeloid derived suppressor cells, immunosuppression, immune regulation, epigenetics, homeostasis

## Abstract

In recent years, most of our knowledge about myeloid derived suppressor cells (MDSCs) has come from cancer studies, which depicts Yin side of MDSCs. In cancer, inherent immunosuppressive action of MDSCs favors tumor progression by inhibiting antitumor immune response. However, recently Yang side of MDSCs has also been worked out and suggests the role in maintenance of homeostasis during non-cancer situations like pregnancy, obesity, diabetes, and autoimmune disorders. Continued work in this area has armored the biological importance of these cells as master regulators of immune system and prompted scientists all over the world to look from a different perspective. Therefore, explicating Yin and Yang arms of MDSCs is obligatory to use it as a double edged sword in a much smarter way. This review is an attempt toward presenting a synergistic coalition of all the facts and controversies that exist in understanding MDSCs, bring them on the same platform and approach their “Yin and Yang” nature in a more comprehensive and coherent manner.

## Introduction

Originated from common hematopoietic progenitor cells, myeloid derived suppressor cells (MDSCs) encompasses a heterogeneous population of immature and mature myeloid cells possessing immunoregulatory activity. These cells suppress both the arms of immune system: innate and adaptive immunity of an individual. In normal and healthy individuals, immature myeloid cells (IMCs) get originated in bone marrow and further differentiate in mature granulocytes, macrophages or dendritic cells. Partial blockage of IMCs differentiation results in the discovery of MDSCs during various pathological conditions reported so far like cancer, trauma, stress, chronic inflammatory state. Substantial evidences suggest the importance of MDSCs in contribution to human malignancies, tumor progression, and metastasis. These cells play a key role in immunosuppression induced by tumor by inducing a state of tolerance (1, 2).

Most of our knowledge till date is mostly based on tumor models and cancer patients, but consistent interest of different fields in this area focused the relevance of these cells in non-cancer situations where over activated immune system need to be suppressed and wherein MDSCs proved as a boon for maintaining homeostasis in immune regulation (3). Due to the phenotypic and functional heterogeneity, the exact mechanism how MDSCs develop, accumulate, and function is still unveiling. Although originating from same precursor cells, they are known to suppress other cells of the same lineage. Possible epigenetic signatures involved in the above phenomena are also covered in this review. What epigenetics has to say about MDSCs recruitment and function is an emerging field of interest for scientists.

Here our alluring goal is to review all the aspects of MDSCs that can give us a better clue to therapeutically target them in cancer and promote them in non-cancer situations. The cross-talk of the signaling pathways involved in the proliferation and accumulation of these cells has been discussed at length.

## Ontogeny and phenotypic characterization of MDSCs: appearances do matter!!

Originated from hematopoietic stem cells, “myeloid progenitors” or immature myeloid cells (IMC) migrate and get differentiated to mature granulocytes, macrophages or dendritic cells. It was in early 70's when accumulation of immune suppressive myeloid cells was associated with tumor progression. Till 2007, before the term ‘myeloid derived suppressor cells (MDSCs) were coined, these immature cells were identified as: “immature myeloid cells (IMCs),” ‘myeloid suppressor cells (MSCs) (4). Similar cells were later on reported in various other forms of cancer. Tumor produced vascular endothelial growth factor (VEGF) acts as a chemo attractant for these cells (3, 5). Transplanted tumors in mice also induced the production of these cells (6, 7) with similar activity of inhibiting antigen dependent T cell activation (8).

Perplexing phenotypes of MDSCs provides both opportunities and frustrations for scientists. Phenotypic characterization of MDSCs became possible because of flow cytometry that senses distinguished cell surface markers on them. The phenotypic variability is dependent on the physiological as well as anatomic site of mice or human. Initially MDSCs in mice were defined as cells expressing cell surface markers Gr-1^*^CD11b+ but lack the typical expression of mature macrophages and dendritic cells. MDSCs in mice are categorized in two major subsets: (i) Granulocytic MDSCs (G-MDSCs) having CD11b^+^ Gr-1^+^Ly6G^+^Ly6C^−^ cells surface markers and ii) Monocytic MDSCs (M-MDSCs) that present markers like CD11b + Gr-1 +Ly6G ^−^ Ly6C ^+^ on their cell surfaces (4, 9, 10). Unlike mice, human MDSCs are characterized as CD11b^+^CD33^+^HLADR^−^. Furthermore, CD15^+^population correspond to G-MDSCs, and CD14^+^ population correspond to M-MDSCs in addition to CD11b^+^CD33^+^HLADR^−^ (11– 16). Recently, a group discovered Lectin type oxidized receptor-1 (LOX-1) in humans as a new marker present on G-MDSCs. Further confirmation in mouse, blood, and spleen is needed for a unifying concept (17).

Because of the ambiguity in the expression of surface markers, many subsets of MDSCs do not exactly get classified into G/M MDSCs. Recently, a novel subset of MDSCs: fibrocytic MDSCs (F-MDSCs) appear to express CD11b^low^CD11c^low^CD33^+^IL-4Rα^+^ on their surfaces and might express HLA-DR unlike other human MDSCs (18–21). Although F-MDSCs show immunosuppressive behavior, but little is known about its differentiation and immunosuppressive mechanism (19). A drive that differentiates common progenitor cells into different subtypes of MDSCs and whether these MDSCs are really different from mature monocytes and neutrophils is a big question nowadays.

Available literature now gives a conclusive picture based on evidences like: (i) MDSCs have an intrinsic characteristic of immunosuppression (ii) in spite of growing mature monocytes and neutrophils with PAMPs, DAMPs or proinflammatory cytokines, they fail to show immunosuppressive activity (iii) MDSCs show distinctive genomic expression profile when compared to neutrophils (22). Inspite of these evidences, it still remains a question and needs to be further explicated.

## Immunosuppressive mechanisms: a different approach

MDSCs are immunologically activated cells. Apart from phenotypic heterogeneity, MDSCs display functional heterogeneity as well. The hallmark of MDSCs is their skill to suppress T cell and NK cell responses, inducing a state of tolerance. Interaction of MDSCs and T cells is not unidirectional where MDSCs control immune response and activation of T cells; and T cells affects MDSCs proliferation and suppressive behavior. Various T cell proliferation and activation suppressive mechanisms are known till date. Different subsets of MDSCs adopt different and specific strategies for exerting immunosuppressive activities. MDSCs produce factors like ROS, iNOS, Arginase-1, and IL-10 to combat against effector T cell responses. In this section, we will be discussing on the different mechanisms adopted by these cells to subdue our immune system. They are known to suppress our T cells in both antigen specific as well as antigen non-specific manner. Following section will let us know every possible mechanism these cells can undergo for immune surveillance.

### G-MDSCs mediated immunosuppression

Enough evidences support the fact that different subsets of MDSCs adopt different mechanisms for immunosuppression. Likewise, G-MDSCs (i) use ROS as a tool for immunosuppression which requires more close cell to cell contact hence supports Ag specific T cell suppression (4, 11, 23–28). It disrupts the TCR/MHC complex formation (11, 16, 23). ROS when interacts with NO, results in the formation of PNT. PNT induces apoptosis of T cells by nitrating the TCR (11, 28, 29).

### M-MDSCs mediated immunosuppression

In contrast to G-MDSCs, physical interaction is lessened in M-MDSCs and suppression is mainly dependent on iNOS, Arginase-1, and IL-10: (i) As arginine is a key nutritional substrate for T cell proliferation, Arginase-1 depletes arginine, producing urea, and ornithine (ii) iNOS produces NO and citrulline utilizing arginine and eventually inhibits T cell proliferation(30–32) (iii) iNOS induced NO downregulates JAK/STAT pathway resulting in T cell apoptosis (iii) IL-10 induces activation of Foxp3+ T reg cells, induce anti-inflammatory M2 macrophage differentiation and expansion of MDSCs population (31, 33–35). NO has a longer half-life than ROS and needs less closer contact, thus M-MDSCs act in a humoral way and suppress non-specific T cell responses.

### F-MDSCs mediated immunosuppression

The exact mechanism of differentiation and immunosuppression is not well-known in recently established subset of MDSCs. But it is supposed that acting through IDO (indoleamine oxidase); it induces T reg population and M2 macrophages population for T cell suppression (20, 21).

## Expansion, activation and migration of MDSCs

### Signal initiators/cytokines/signal initiation

Our immune system has evolved mechanically to protect us from the deleterious effects of inflammation. One such strategy adopted by our immune system is the generation of immune suppressive cells like MDSCs from myeloid progenitors that offsets T cell action. The expansion, activation, and migration of these cells should be precisely synchronized reason being, as stated above they can be boon at one time and curse at the other. Several growth factors/cytokines are involved in MDSCs expansion, activation, and migration in both humans and mice, including M-CSF, GM-CSF, G-CSF, IL-13, IL-1ß or IL-6, TNF-α, VEGF, PGE_2_, proinflammatory S100 proteins, C5a, LPS, PARP-γ, HSP72, Flt3L. They can act independently or in a synergistic manner. Depending on the concentration and microenvironment, they can be stimulatory and inhibitory mediator of immune system.

Discussing about the relevance of these growth factors, several animal studies has come up with beautiful results mainly in tumor model. G-CSF^−/−^ or G-CSFR^−/−^ mice turned neutropenic, while anti-G-CSF treatment was effective in inhibiting MDSCs infiltration near tumor site. Exogenous G-CSF administration results in recruitment of MDSCs and shuts down innate immune system. A direct association is already established between G-CSF and G-MDSCs in tumor bearing mice (18). Where induction of G-CSF leads to tumor growth, it can act as a blessing during pregnancy and other pathological conditions which requires enough G-MDSCs to inhibit our innate immune system for leading a healthy life. Likewise, knockout studies for other cytokines like IL-1, M-CSF evidently provide a glimpse why and how these growth factors are important for MDSCs expansion, activation and migration (36).

Not just the cytokines are indispensable elements for MDSCs development, but the way they act is more important. Two theories have been proposed for expansion, activation and migration of MDSCs in tumor model: (i) “**One signal hypothesis**” states that one signal is enough for the differentiation of hematopoietic progenitor cells into MDSCs (ii) “**Two signal hypothesis”** states that the differentiation of HSC to MDSCs occurs in two steps: (i) HSC-IMC transition via STAT-3 signal on activation with cytokines like G-CSF,GM-CSF,IL-6,PGE2 and (ii) IMC-MDSCs transition mediated by mainly proinflammatory cytokines LPS, S100 proteins, IL-1 ß etc. and activating NFκ-B, PI3K, and STATs mediated signaling.

### Signaling and crosstalk

**Figure 1:**
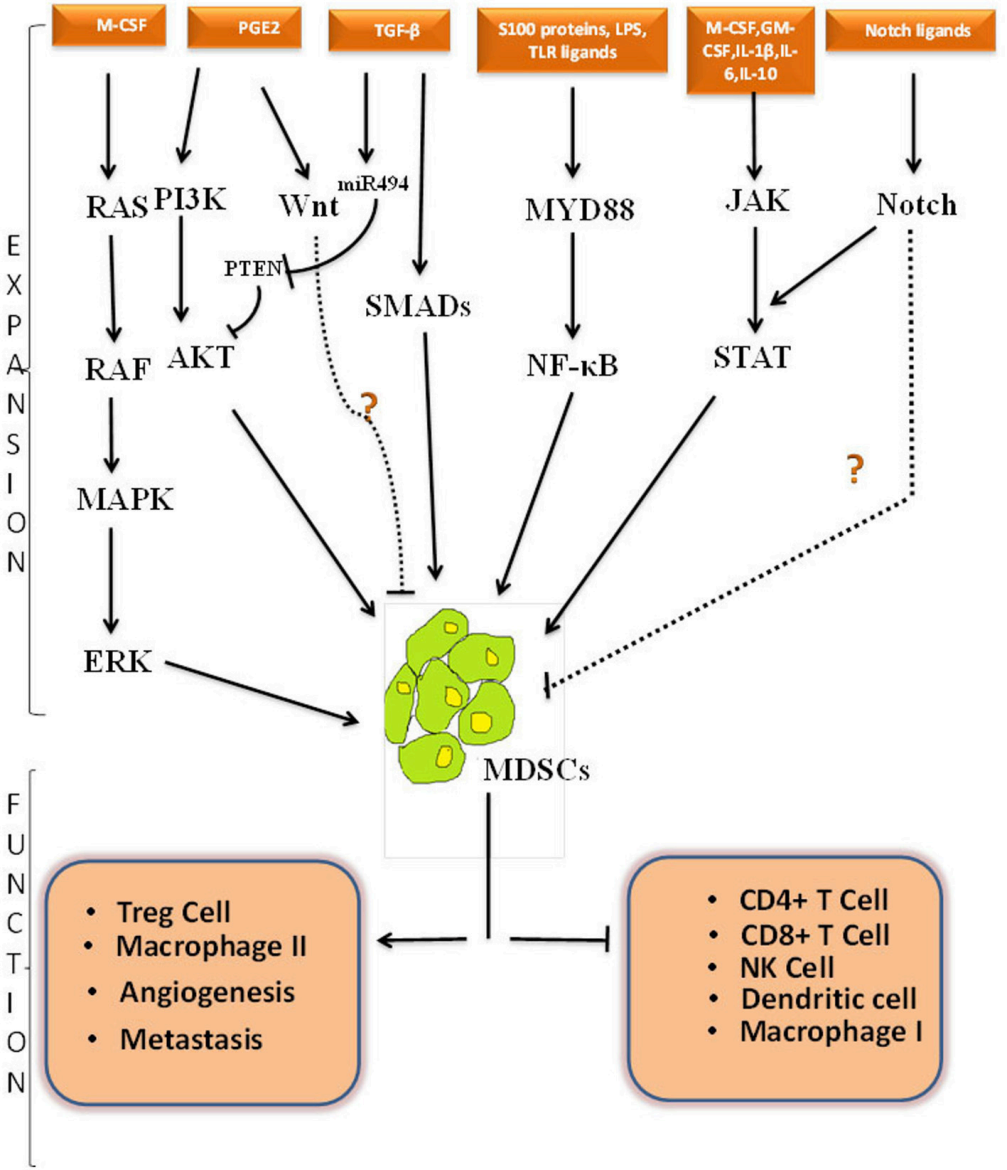
Cross-talk among different signaling pathways involved in MDSCs expansion, activation and function. Schematic representation of possible pathways involved in MDSCs expansion, activation and function. Black solid line represents known pathway for MDSCs proliferation whereas black dotted line represents predicted pathway and crosstalk that can be involved in MDSCs regulation. How NOTCH pathway and Wnt pathway directly regulate MDSCs expansion is shown with a question mark. The positive and negative regulatory function of MDSCs is depicted in lower part of figure. PGE2, Prostaglandin E2; M-CSF, Macrophage colony stimulating factor; GM-CSF, Granulocyte-macrophage colony stimulating factor; IL-1β, Interleukin 1 Beta; IL-6, Interleukin 6; IL-10, Interleukin 10; LPS, Lipopolysacchharide;TGF-β, Transforming growth factor Beta; PI3K, Phosphoinositide 3-kinase; AKT-Protein kinase B, a serine threonine specific protein kinase; PTEN, Phosphatase and tensin homolog; MAPK, A mitogen-activated protein kinase; ERK, Extracellular Signal-regulated Kinase; JAK, Janus kinases; STAT, Signal transducer and activator of transcription; TLR, Toll like receptor; Myd88, Myeloid differentiation primary response 88; NF-κβ, nuclear factor kappa-light-chain-enhancer of activated B; NK, natural killer cells; IFN-γ, Interferon gamma; Treg, T regulatory.

Although the exact signaling and molecular mechanism involved behind the generation of MDSCs is still evolving, it is believed that dysregulation happens somewhere in the canonical signaling pathway of myeloid development. We will be discussing the crosstalk among different signaling cascades initiating from the cytokines and how these growth factors independently or synergistically control the activation of MDSCs in different biological conditions. Targeting these pathways may clarify the mechanisms and help us in expanding or abolishing MDSCs when and wherever required. Schematic representation of different signaling cascades is elucidated in Figure 1.

#### Ras signaling

Ras proteins are considered indispensable components of signaling pathways stemming from cell surface. Ras signaling has an important role to play in myeloid development. Ras protein cycles through “on” and “off” state depending on the binding of GTP and GDP, respectively. Under physiological states, this transformation is regulated by guanine nucleotide exchange factors (GEF) that promotes Ras activation and GTPase activating proteins (GAP) that accelerates Ras mediated GTP hydrolysis. Ras drives cell proliferation, survival and migration thus promoting tumors to attain a malignant phenotype via autonomous and non- autonomous mechanisms. Over activated mutant Ras alters proliferation and differentiating potential of macrophages. Oncogenic Ras promotes angiogenesis via VEGF and creates an immunosuppressive state by adopting methods to evade host anti-tumor response like: (i) reduced expression of MHC on cancer cells (ii) educating stroma to recruit macrophages or MDSCs at TME (37–40). Induction of MDSCs recruitment through Ras signaling via cytokine M-CSF is well-known in cancer but need to be explored in other pathological conditions where Ras signaling plays crucial role in immune surveillance during disease development.

#### PI3K/AKT signaling

A huge heterogeneity is present for the signaling proteins that binds to the phosphorylated phosphatidylinositol and based on these substrate specificities, PI3K has been divided into several classes. Cell type of the immune system determines which member of PI3K will be activated. Cytokines like PGE2, IL-2, IL-6, IL-7, GM-CSF, and interferons that regulate MDSCs development activate one of the classes of PI3K. Cytokines mentioned here have the potential to activate more than one signaling thus definitely a cross talk exists between PI3K/AKT signaling and other signaling cascades. A recent report proposed an unexplored connection between PI3K/AKT and Wnt signaling discussed in detail below. A clue for PI3K/Akt mediated MDSCs regulation prompted other researchers to look into its signaling in detail. Selective disruption of various isoforms of PI3K showed altered phagocytic and chemotactic activity *in vivo*. Knock out studies in mice further validated the same in neutrophils (41). As regulating other cells, PI3K have the possibility to modulate MDSCs function as well. A study in aging mice with a defect in PI3K/Akt signaling contributed to compromised immune system and showed MDSCs accumulation in bone marrow and secondary lymphoid organs (42). The negative regulators of PI3K, i.e., SHIP (SH2 domain containing-5-inositol phosphatase) and PTEN (phosphatase and tensin homolog) controls phosphoinoside metabolism in immune cells (43). Therefore, increasing the activity of SHIP may be beneficial in certain diseases like cancer, trauma, viral load or infection and harmful in situations where MDSCs are desirable for immune surveillance like pregnancy, obesity and diabetes. Based on the literature reported till date, PI3K pathway could be one of the major pathways involved in the regulation of MDSCs.

#### JAK/STAT signaling

Inflammation is the complex state of biological response where immune system of our body acts against the invading pathogens. The Janus kinase/Signal transducer and activator of transcription (Jak/Stat) pathway is well-known to regulate the inflammatory response. Jak family consists of four members: Jak1, Jak2, Jak3, and tyrosine kinase2 (Tyk2). Stat family is composed of seven members: Stat1, Stat2, Stat3, Stat4, Stat5a, and Stat5b. Jak could be activated by cytokine of IFN family members: α, β, γ. Stat after getting phosphorylated and activated by Jak, translocates to the nucleus and acts as a transcriptional regulator. Few studies have shown Stat gets stimulated by M-CSF that is already stated as a growth inducing factor of MDSCs. So there must be some connection between activated Stat and MDSCs accumulation. MDSCs interfere with host immune system by inhibiting immune cell responsiveness toward IFN cytokines in tumors (44). Though Jak phosphorylation and Stat activation occurs through a variety of cytokines, but some specificity is still there depending on the type of immune cell and the isoform of Stat. In cancers, Stat along with NF-κB facilitates signal transduction coming from extracellular stimuli. Among all Stats, Stat3 plays a very crucial role in incidence of cancers. How this molecule regulates MDSCs activity by Arginase 1 was very well explained by Vasquez-dunddel et al. (45) where they reported that Arginase promoter has multiple binding sites for Stat3. si-RNA against STAT3 abolished MDSCs activity. A number of studies reported that constituent activation of Stat3 promotes inflammation and tumor growth by expanding MDSCs population by upregulating Stat3 target genes like B-cell lymphoma XL(BCL-XL), cyclin D1, Myc, and survivin (46–49). Being itself activated by proinflammatory cytokines like IL-4 or IL-6, Stat3 can downstream affects proinflammatory proteins like S100 family proteins (50). Although the precise mechanism is not yet elucidated but it is postulated that overexpression of S100 family proteins i.e., heterodimer of S100A8/S100A9 assisted NADPH oxidase complex formation which generates ROS and interferes with the differentiation of myeloid cells (28). An autocrine signaling exists as MDSCs also has receptors for S100 proteins on their cell surface and in turn these proteins help MDSCs migration toward TME. The mechanism needs further attention, as their signaling will definitely provide a link between inflammation and immune suppression theory. Other factor that negatively regulates Stat3 is IRF8 (Interferon regulatory factor 8). IRF-8 when overexpressed in mice lead to the reduction in MDSCs, acting as a negative regulator of Stat3 and thus MDSCs (51). So targeting Stat3 could be a therapeutic potential in cancers or wherever immunosuppression is harmful.

#### TGFβ and PGE2/COX signaling

Talking of TGFβ, an effective regulator of inflammatory response operates by affecting the activity of innate and adaptive immune cells. Depending on the cellular context and target genes, it can be a positive and negative regulator of transcription. TGFβ can regulate MDSCs proliferation via microRNA expression. A study showed miR494 deletion in MDSCs attenuated tumor growth. Still the exact signaling initiating from TGFβ involved in MDSCs expansion is not known in biological conditions apart from cancer.

Synthesized by COX-2, PGE2 possess both proinflammatory and immunosuppressive property. PGE2 signals through PGE2 receptor E-prostanoid (EP) in MDSCs (52, 53). Agonists of EP receptor including PGE2 stimulated bone marrow stem cells to generate MDSCs. Inhibition and *in vivo* administration of COX-2 could significantly restore the differentiation of BM cells and reduce MDSCs accumulation, respectively (54). In total, we can say that PGE2 and COX-2 synergistically regulate the function and differentiating potential of MDSCs.

#### Recent studies concerning MDSCs regulation

##### Notch signaling

It is well-established that Notch signaling regulates differentiation and functions of myeloid derived cells like DC, macrophages and mesenchymal stem cells (55, 56). In recent years, pleiotropic function of Notch has come up, where Notch is reported to modulate the immune responses by activating different immune cells. How Notch-RPB-J regulates MDSCs immunosuppressive behavior is explained by gain of function and loss of function experiments which proves that blockage of Notch pathway promoted the expansion of MDSCs with low immunosuppression (57). They unambiguously painted the regulatory axis of Notch Signaling as: Notch-IL6-STAT3-MDSCs. However, a lot more questions need to be addressed.

##### Wnt signaling

A well-established interaction between tumor and stroma is mediated by factors released either by tumor or by stroma. Tumor cells educate the stroma to recruit and maintain heterogeneous population of immature cells like MDSCs to potentially suppress T cell responses and promote tumor growth (47). Wnt pathway has been shown to antagonize differentiation of MDSCs and support the differentiation of mature DCs. β catenin should be downregulated in MDSCs for them to get accumulated in mice as well as humans (58). But still a question rose, what drives downregulation of β catenin in MDSCs. Is something to do with stroma? And the answer was yes. A protein Dickkopf-1, inhibitor of β catenin dependent Wnt signaling is highly expressed in cancer cells and apart from its basic function, it inhibits β catenin and promotes MDSCs accumulation (59, 60). Dysregulated β catenin has been reported in many cancers but another study supported the above concept where PLCγ2–/– MDSCs display reduced β-catenin, and overexpression of β-catenin lessens tumor growth (58).

Wnt signaling has so much to do with human trophoblast invasion and differentiation (61). It is also reported to play role in human fetal growth in first and second trimester. How Wnt regulates MDSCs activity during pregnancy still remains a question of interest for researchers.

## Epigenetic control of MDSCs

In spite of originating from same population of cells, MDSCs keep a distinct ability to suppress other immune cells. It gives us a faint clue of changes in epigenetic signatures. Epigenetic mechanisms play a crucial role in gene expression and cellular differentiation. It defines all heritable modifications without any alteration in DNA sequence. DNA modifications, histone modifications and RNA interference initiates and sustain epigenetic regulatory network.

### DNA modifications in MDSCs

One of the most important DNA modifications is DNA methylation that mediates gene silencing with transcription machinery. DNA methyltransferases (DNMTs) aids both *de novo* and inherited DNA methylation which transfers methyl group to 5′position on cytosine residues with CpG islands (62). How DNA methylation regulates MDSCs expansion and biological activity is well-studied with the administration of Δ9-tetrahydrocannabinol (THC), a potent inducer of MDSCs. It enhanced promoter methylation of DNMT3a and DNMT3b and rescues arginase-1 and Stat3 expression (63, 64).

### Histone modifications in MDSCs

A form of epigenetic regulation where covalent modifications like acetylation, phosphorylation or ubiquitination alters the histone core structure and affects the binding efficiency of “effector molecules” on the DNA sequence. The best studied modification is acetylation. A dynamic balance between acetylation by HATs (histone acetyltransferases) and deacetylation by HDACs (histone deacetyltransferases) affects the gene expression (65). Does HDAC have any role to play in MDSCs expansion and activation impelled scientists to work in this area. Rosborough BR in 2012 reported that *in-vitro* or *in-vivo* administration of a naturally occurring antifungal metabolite TSA produced from Streptomyces having HDAC ability expands M-MDSCs in NOS^−^ and heme oxygenase (HO) ^−^ dependent manner (66). A new member of histone deacetylase family, HDAC11 seemed to serve as gatekeeper of myeloid differentiation and acts as a negative regulator of MDSCs expansion (67). Reports are there where mere inhibition of retinoblastoma gene via HDAC2, another histone deacetylase promoted switch of M-MDSCs to G-MDSCs in cancer, but lack immunosuppressive activity (68).

Epigenetic modifications may play a significant role in regulation of one of the most important transcription factors Stat3 in promoting MDSCs expansion and activation. Whether it regulates by phosphorylation or ubiquitination is not yet known. A recent study stated that p66, a component of Mi2/NuRD/HDAC complex suppresses Stat3 phosphorylation (Y705) and ubiquitination (K63) by directly interacting Stat3 (69). Any regulatory mechanism controlling Stat3 activation will definitely be a new therapeutic potential against few pathological complications.

### miRNA regulation of MDSCs

In physiological conditions, miRNAs are well-known to regulate gene expression involved in cell development and differentiation. Emerging literature coming up with the idea that miRNAs are vitally involved in proliferation, development, migration and function of MDSCs.

MiR-210, miR-9, miR-690, miR-494, miR-155, miR-21, miR181b, miR-34a are known to epigenetically modify several promoters or genes and enhance immunosuppression mediated by MDSCs whereas miR-17-5p, miR-20a, miR-223, miR-146a, miR424, suppresses the suppressor (Table 1).

**Table 1:** Epigenetic regulation of MDSCs.

	**Epigenetic modulation**	**Target gene/pathway**	**Species**	**References**
**1**	**DNA MODIFICATIONS**			
a	DNA methylation	JAK/STAT	Mice	(63, 64)
**2**	**HISTONE MODIFICATIONS**			
a	**Acetylation**			
	HDAC2	Retinoblastoma	Mice	(68)
	HDAC11	Not mentioned	Mice	(67)
**3**	**POSITIVE REGULATION OF MDSCs (miRNAs)**			
a	miR-210	Arginase-1, CXCL12, IL-16	Mice	(70)
b	miR-9	Runt-related transcription factor-1	Mice/Human	(71)
c	miR- 494	PTEN/AKT	Mice	(72)
d	miR-690	CCAAT enhancer binding protein	Mice	(73)
e	miR-155, miR-21	SOCS/SHIP-1/PTEN	Mice	(74)
f	miR-17-5p and miR 20a	SHIP-1/PTEN	Mice	(75)
g	miR-181b	CYL D,NF-KB	Human	(76)
h	miR-34a	N-myc	Chimera	(77)
**4**	**NEGATIVE REGULATION OF MDSCs (miRNAs)**			
a	miR-223	Myocyte enhancer factor-2(MEFC-2)	Mice	(78)
b	miR-424	PU.1/NFI-A	Human	(79)
c	miR-146a	TRAF6/NF-KB/IRAK1	Mice	(80)
**5**	**NEGATIVE REGULATION OF MDSCs (siRNAs)**			
a	A20 siRNA	A20	Mice	(81)
b	STAT3 siRNA	STAT3-arginase-1	Human	(82, 83)
c	SCF siRNA	Stem cell factor	Mice	(84)
d	CK2siRNA	CK2-NOTCH	Mice	(85)

### siRNA regulation of MDSCs

Artificial double stranded RNA of nucleotide length 20–25, acts as a transcriptional regulator and trigger gene silencing. Several siRNAs are targeted to regulate MDSCs expansion, differentiation and activation e.g., A20si-RNA, Stat3si-RNA, Stem cell factor si-RNA, Casein kinase 2 si-RNA either induce apoptosis or promote differentiation of myeloid cells. These all strategies could improve immune therapy for treating advanced cancer or maintaining maternal-fetal tolerance during pregnancy. The mechanism how these siRNAs regulates MDSCs still remains an unanswered question.

## Yin and Yang arms of MDSCs

When an antigen enters our body, inflammation occurs and subsequently innate immune system deals with it. Antigen presented by innate immune cells with respective MHCs recruits other adaptive immune cells for clearance of invading antigens and if immune system fails to complete the clearance of antigen successfully i.e., the host immune system is hijacked and misguided by the antigens, the situation creates an intricate mess of immune cells that is referred as chronic inflammation. To suppress the transition of acute to chronic inflammation, a negative feedback loop is required. This negative feedback is where **Yang** interaction of MDSCs comes into play. MDSCs have an inherent capability of immunosuppression either in acute or chronic inflammatory condition. Infiltration of immunosuppressive cells (MDSCs) at the site of inflammation aids in the suppression of transition from acute to chronic state. In a normal healthy individual, a balance of MDSCs and anti-inflammatory cytokines is in homeostasis with pro-inflammatory cytokines. Any imbalance in this immune homeostasis may lead to the occurrence and development of disease.

The differential behavior of MDSCs in acute and chronic inflammation is not anew. It behaves as double edged sword; in acute or regulated inflammatory situations, on one hand their accumulation beneficially lessens the burden of disease thus showing “**Yang” behavior** and on the other hand accumulation or depletion of MDSCs in dysregulated or chronic inflammatory settings enhances the burden of disease and show **“Yin” behavior**. The Yin role of MDSCs is to suppress our immune system up to a certain extent favoring the disease progression.

We need to discuss and draw a comparative picture of MDSCs and Tregs cells in parallel for having better insights into the immunosuppressive behavior of these cells. Treg cells are known for immunosuppression by restricting the proliferation and activation of effector T cells. Qiao et al. have very well-explained the Yin and Yang of T reg cells in autoimmune diseases (86). They discussed the pathogenesis of autoimmune disorder (AD) occurrence due to the breakdown of immune homeostasis via Tregs (Yin) whereas maintenance of immune homeostasis (Yang) in healthy individual which in retrospect provided the coherent articulation of understanding similar merits in MDSCs as well.

**Figure 2:**
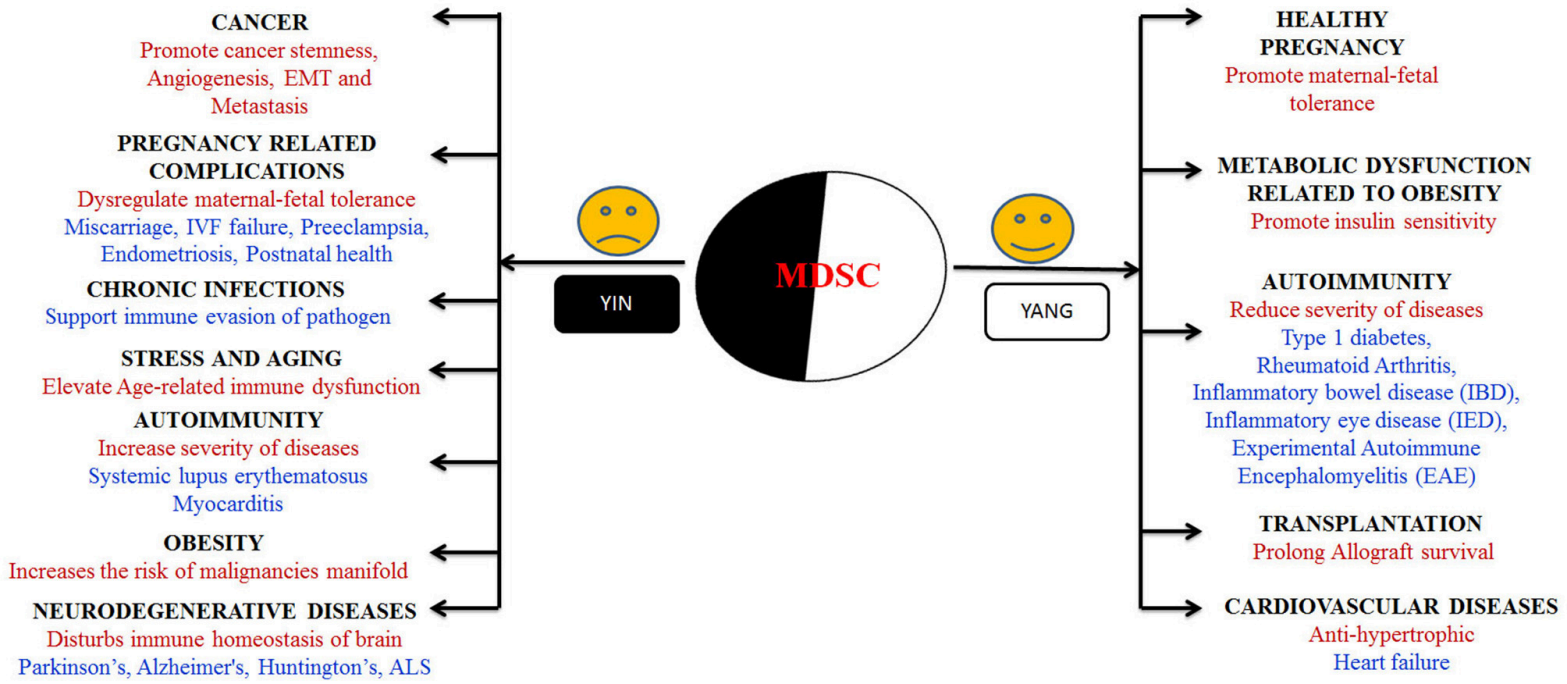
Graphical representation of “Yin” and “Yang” arms of MDSCs in various biological conditions. “Yin” arm presents the diseases where immunosuppressive behavior of MDSCs augments the disease progression whereas “Yang” presents the array of diseases and conditions where the presence and immunosuppressive behavior of MDSCs lessens the disease burden.

MDSCs are reported to change their fate and activity according the environment they are exposed to (87, 88). In one hand, they potentially enlighten the milieu of pregnancy, diabetes and prevent allograft rejection (89–91). On the other hand, tumor microenvironment (TME) promotes MDSCs accumulation that is a major obstacle for natural anti-tumor immunity and enhances tumor growth (92). These cells received much attention in recent 8–10 years due to its functional heterogeneity and outstanding immunomodulatory nature; hence they can be used as a new potential therapeutic target in different biological conditions. In this section, we tried to discuss what happens and why MDSCs showing their yin and yang role with special emphasis of which subset present in different biological conditions (Figure 2).

### Yin and Yang role of MDSCs in pregnancy and related complications

The immune system of a pregnant female need to be modulated in a very well regulated way so that without compromising her own optimum immunity level, her immune system should simultaneously protect the semi-allogenic fetus from invading pathogens and confer tolerance from rejection. It has long been assumed that the maternal immunity must be compromised or redirected in a way to ensure a successful implantation and pregnancy. This state of modulation in maternal immune system is achieved by the synergistic interplay of regulatory cells like MDSCs and T regs.

Kostlin et al. reported that MDSCs especially G-MDSCs are significantly increased in healthy pregnant women during all stages of pregnancy compared to non-pregnant controls, implying their possible role in pregnancy (91). MDSCs are not only reported to regulate maternal fetal tolerance during pregnancy but are also well-known for T cell modulation and necessary for the control of inflammation. In line with the above study, our lab reported increased MDSCs in the first trimester compared to the third trimester of healthy pregnant women, proving its importance during implantation (93). On being investigated at the mechanistic aspect, MDSCs utilize multiple mechanisms to silence our overactive immune system during pregnancy: T cell anergy via secretion of ARG-1, iNOS, IDO, induction of other immunosuppressive cells (Tregs, uNK) and *in-utero* inflammation maintenance (94, 95). The significance of MDSCs showing the “Yang” role in healthy pregnancy tempted researchers to investigate their role in pregnancy related complications in the last few years.

Our lab works in the same direction and parallely reported that functionally suppressive MDSCs (CD11b+CD33+) are reduced in blood and endometrium of miscarriage patients (93). In another recent study, G-MDSCs were downregulated in IVF failures patients as well (96). A very recent literature showed pregnancy associated G-MDSCs and effector molecule Arg-I is significantly inhibited in pre-eclampsia patients with no difference in the population of Treg cells (97).

The scenario of pregnancy where MDSCs beautifully portray the Yang behavior, we cannot ignore the other side of the coin. Recent study in endometriosis reported elevated level of G-MDSCs as well as its immunosuppressive activity (ROS, Arginase I, suppress T cell proliferation) exaggerates endometrial lesions. This study was further confirmed when depletion of MDSCs with Gr-1 antibody in mice dramatically decreased endometrial lesions (98). Elevated level of G-MDSCs in cord blood of preterm born infants, modulating T cell responses suspects their role in regulating postnatal immunity dysfunction (99). The circulating levels of G-MDSCs are elevated in pre-term born neonates, modulate T cell responses and are necessary for control of inflammation (100). Till date, the studies discussed above corroborate Yin behavior of MDSCs in various gestational disorders, where elevated MDSCs disturb the immune homeostasis and helps in the development of disease. These noteworthy findings anticipate an important concept: Immune homeostatic milieu is essential for successful pregnancy. The breakdown of this homeostasis exposes the Yin behavior of MDSCs. Hence, these reports are fascinating researchers all around to shed light on these cells for exploring its regulatory nature in normal pregnancy vs. reproductive failure.

### Yin and Yang role of MDSCs in cancer

Most of our knowledge about MDSCs till now comes from cancer studies. The presence of MDSCs with immunosuppressive activity is widely evident within the cancer sites since early 1900s. MDSCs not only promote tumor angiogenesis and invasion, but also impair anti-tumor innate and adaptive immune response; they are likely to subvert host immune surveillance; enhance the stemness of cancer cells and modifies pre-metastatic niche (11, 101–107). Accumulation of MDSCs in different tumors at tumor microenvironment induced by various chemokines is well-known but recently secretory IgM secreted by B cells is reported to accumulate MDSCs in chronic lymphocytic leukemia (CLL) (108). These studies suggest that MDSCs which was historically recognized as T cell suppressors; actually have additional potency to orchestrate more strategies for tumor progression and metastasis. Hence several cancer studies and tumor models present the “Yin” role of MDSCs where accumulation, expansion and activation of MDSCs create a permissive environment most suitable for the growth of malignant cells (106). Subset M-MDSCs are found to be remarkably high in multiple myeloma, leukemia hepatocellular carcinoma, prostate cancer and melanoma, while G-MDSCs are prominent in renal, colon, lung, breast and pancreatic adenocarcinoma (1, 109). Known to act solely as negative regulators of immune system, the Yang side of MDSCs is not yet reported as per our knowledge. Controversies do exist for the complex regulatory behavior of MDSCs in cancer at different stages. Most of the studies positively correlated the frequency of MDSCs with the cancer stage (110–112) while MDSCs are reported to be in inverse relation to the advanced stages of cancer. Moreover, contrary to other reports, tumor infiltrating MDSCs show reduced immunosuppressive behavior when compared to peripheral MDSCs (103). The comparative behavior of MDSCs in benign and malignant cancer still remains a hotspot research area which needs further exploration. This might clarify the discrepancy present in correlating MDSCs with stages of cancer. An immense focus is needed to reach to a unifying concept before inclusion of therapeutic targets into clinical practice (113).

Treatment of cancer is a challenging issue for both scientists and clinicians. Common cancer treatments known to us include surgery, radiotherapy, chemotherapy, and endotherapy. Targeting MDSCs via radio or chemotherapy is effective upto a limited extent but fail to provide long term benefits. MDSCs may weaken the anti-tumor effect of cancer vaccines but effective response of vaccines increases when used in conjunction with MDSCs inhibition. They collectively adopt different mechanisms for MDSCs inhibition: (i) deactivation (PDE5, histone deacetylase, NO inhibitors, Arginase inhibitors, ROS inhibitors, STAT3 inhibitors) (ii) inhibition of recruitment at the site of tumor (CCR5 antagonist, CCL2 inhibitor) (iii) differentiation (ATRA, Vitamin A, D3) (iv) Regulation of myelopoiesis and depletion (Tyrosine kinase inhibitors, cytotoxic agents, anti Hsp90) (107, 114, 115). Recently, liver-X nuclear receptor (LXR) agonism i.e., GW3965/RGX-104 and a long non-coding RNA i.e., Lnc-chop are also used to regulate immunosuppressive function of MDSCs as well as its abundance in tumor environment (116, 117). Targeting MDSCs still remains a challenging task because of the heterogeneity and lack of specific marker (118, 119). Immunotherapy is another promising and encouraging strategy for cancer treatment. Immune checkpoint inhibition immunotherapy is popular therapy now-a-days with low toxicity levels and provides long term survival benefits. As, immune check points are basically involved in maintenance of self-tolerance, assistance and regulation of immune response. Immune checkpoint therapies (ICTs) act on both the stimulatory or inhibitory pathways and activate the immune system against cancer. The most common inhibitory pathways are programmed death cell protein-1/programmed death cell ligand-1 [PD-1/L1] and cytotoxic T lymphocyte antigen-4 [CTLA-4] and stimulatory pathway are OX40 (TNFR; Tumor necrosis factor receptor), Inducible co-stimulator (), Glucocorticoid-induced TNF receptor family-related protein (GITR), 41BB (CD137), CD40. Nivolumab, Pembrolizumab, and Ipilimumab are the antagonist agents used as immune checkpoint inhibitors. MOXR-0916, AMG-228, JTX-2011, Utomilumab, CP-870893, APX005M, ADC-1013, lucatumumab, Chi Lob 7/4, dacetuzumab, SEA-CD40 are the agonist agents used for targeting the stimulatory pathway. Recently, Ipilimumab have been used for treatment of melanoma cancer patients. Ipilimumab directly affects the count and immunosuppressive function of MDSCs. Recent studies also suggest that cancer patients become resistant to antibodies targeted against these immune checkpoints due to the presence of MDSCs at the site of tumor. MDSCs hinder the activity of ICTs so it could be a favorable target during combination therapy. Despite the efficiency of ICTs in unleashing immune system and maintaining self- tolerance, they sometimes pose a threat to a range of auto immune disorders. (120–125). A question rises if ICI therapy can increase the risk of autoimmune diseases, blocking of MDSCs based therapy can also raise the same question. Literature is sparse in this field and no report till today says about the risk of autoimmunity that may occur on blockade of MDSCs expansion, recruitment, activation or MDSCs when combined with ICTs. The need of the hour is to revisit the immunological aspect of MDSCs origin for suitable answer to this query. Question about the varied response of ICTs remains unanswered too. Though promising, but inconsistency in the results did not allow us to reach to a conclusion about ICTs alone or in combination with other drugs (126, 127). Recently, combination strategy has been applied in murine model of renal and lung carcinoma where etinostat enhanced the efficacy of PD-1 targeted immune therapy (120, 128). Further studies are warranted in upcoming years to develop an efficient strategy for the treatment of cancer patients.

### Yin and Yang role of MDSCs in autoimmunity

The involvement and expansion of MDSCs in both human and murine models of autoimmune diseases is clearly evident. Contradictions persist due to varied heterogeneity in terms of action and effect of MDSCs in different autoimmune disorders, a plausible comprehensive theory is still not valid in this disease (129). *In vitro* expanded MDSCs keep the potential to clear inflammatory tissue by regulating and promoting the expansion of other immunosuppressive population of Tregs. They significantly inhibit Th1 and Th17 immune responses and thus the severity of diseases in models of rheumatoid arthritis (RA), inflammatory bowel disease (IBD), inflammatory eye disease (IED), experimental autoimmune encephalomyelitis (EAE), and Type 1 diabetes (130). In all the above mentioned autoimmune diseases, MDSCs outperform as “**Yang**” player of the team. Recently, two drugs, Cannabidiol, Glatiramer acetate are used for the enhancement of MDSCs dependent Tregs cell generation and reduction of pro-inflammatory cytokine secretion in IBD, Multiple sclerosis mice model (131, 132).

Contrasting theories are also present which suggest that there might be proinflammatory angle to MDSCs which conventionally have an anti-inflammatory behavior in almost all cases. In case of RA, a report suggests, that MDSCs have found to portray a rather unconventional pro-inflammatory behavior during the early stages of the Arthritis (133, 134). In addition, a recent citation for behavior of MDSCs in RA also suggests that depletion in the number of MDSCs during the early stages of RA causes a weaker inhibition of T cells thereby causing pro-inflammatory response, which in this case is an indirect representation. Due to the lack of more evidence and other substantial literary representations it still remains unclear whether the anti-inflammatory role has become pro-inflammatory or the shift has been caused indirectly by T-cell due to change in the population of MDSCs(95). Therefore, the immune regulation via MDSCs in RA still remains elusive.

Contrary and apart from Yang nature of MDSCs, they also show “**Yin” behavior** in various autoimmune disorders such as Systemic lupus erythematosus (SLE) and Myocarditis. These cells expand in patient's blood and its frequency is directly correlated with IL-17 producing Th-17 cells and severity of diseases (95). The risk of autoimmunity associated with immune checkpoint therapy used in cancer is also due to the Yin behavior of MDSCs (120, 121). Strong correlations do exist between cancer and autoimmunity that need to be considered further. This correlation might be beneficial in answering the queries related to pathophysiology of autoimmune disorders.

### Yin and Yang role of MDSCs in stress and aging

With age, the immune system gets remodeled: the process termed as immunosenescence. Both the arms of immune system: innate and adaptive gets compromised and a person becomes susceptible to various pathological conditions like cancer, autoimmunity, and infections. Inflammageing: Increase in systemic inflammation is a well-documented feature of aging where the regulatory mechanism of immune system gets hampered. Impairment in responding to stress is another feature of aging. One of the possible reasons of this compromised immunity in elderly people can be our immune suppressive MDSCs (135). Recent publications have positively correlated stress and aging with MDSCs in both aged human and mice but which specific subset of MDSCs is involved here is still a perplexing question. Recent study has shown that both natural and augmented aging in mice drives an expansion of MDSCs through NF-κB dependent pathway (136). In these conditions, increased MDSCs population worsens the disease severity by aggravating type 2 responses and alleviating age-related immune dysfunction (“**Yin”** of MDSCs). The dysregulation of immune homeostasis and poor response to vaccines may be due to the accumulation of MDSCs. Till date, **Yang** role of MDSCs is not seen in aged people. Further research is now needed to target MDSCs for the proper regulation of immune system.

### Yin and Yang role of MDSCs in obesity

Obesity is a chronic low-grade inflammatory disease and is linked with several immunological disorders. Pro-inflammatory milieu in adipose tissue mimics that in tumor microenvironment with the same constellation of molecules. Studies till now states that chronic inflammation of adipose tissue in obese mice/ human drives the accumulation of MDSCs and thus increases the rate of malignancies many fold when compared to lean controls (“**Yin**” of MDSCs)(137–139). Obesity promotes immunosuppressive environment by accumulating MDSCs both at local and systemic level via production of chemokine factor CCL2. Experimental data tells us that M-MDSCs and G-MDSCs are accumulated in obese mice whereas M-MDSCs are elevated in humans (138). Very recently, the “**Yang**” behavior of MDSCs came into consideration when they showed protective role against some of the metabolic dysfunctions associated with obesity in contrast to exclusive detrimental role of tumor induced MDSCs (140). Surprisingly, obesity driven MDSCs decreased blood glucose levels and lessened the burden of insulin tolerance. This unusual behavior of MDSCs in obesity leaves us with questions in our mind asking how they exceled in showing their **Yin and Yang** sides simultaneously. The mechanistic study need to be explored with the aim to counteract its detrimental role over beneficial role.

### Yin and Yang role of MDSCs in transplantation

Treg cells were the key players in controlling allograft rejection till researchers discovered the beneficial and desirable role of MDSCs were discovered in various inflammatory conditions like solid organ transplantation, dipping unwanted immune system(141). Since MDSCs shield tumor antigens from recognition, they are closely associated in induction and maintenance of immune tolerance toward organ transplantation(142, 143). Interacting with network of other immune cells, MDSCs regulate host immune system managing allograft survival (“Yang” of MDSCs) (144). It was widely reported that M-MDSCs accumulated during bone marrow, kidney and heart transplantation whereas the subset of MDSCs remains unidentified in skin transplantation. It has been predicted that MDSCs infiltration toward graft can suppress the incidence or severity of graft-vs.-host disease (GVHD) (145). Recent studies further prove this anticipation. Intestinal Transplant (IT) recipients accumulates MDSCs and suppress T cell immune response toward donor antigen (146). Accumulation of M-MDSCs as well as Tregs is reported to enhance graft survival in Almost Tolerant Kidney Transplant Recipient (ATKTR) individuals (147). A recent report has shown Dexamethasone induced Myeloid-Derived suppressor cells prolong allocardiac graft survival through iNOS and glucocorticoid receptor-dependent mechanism (148). MDSCs based cell therapy aids in prolonging graft survival and transplantation tolerance reflects the “**Yang”** behavior of MDSCs. A tight regulation of cell therapy in terms of time and contact is prerequisite else over activation of MDSCs may result in complications such as infection or tumor development showing the “**Yin”** behavior of MDSCs (141). The Yin and Yang behavior is also dependent on the differential effect of immunosuppressive drugs on MDSCs during implantation. Some drugs significantly augment the function of MDSCs, some diminish and still some remains unexplored. The negative effects of these agents may diminish the tolerance induction (88, 149).

The recent advances and importance of MDSCs has garnered scientists to explore about MDSCs proliferation, differentiation and activation in clinical investigations during allograft transplantation. Studies until recently are there in *in-vitro* models or animal models, and it gives a platform for researchers to explore the mechanism involved in tolerance induction.

### Yin and Yang role of MDSCs in chronic infections

On the attack of pathogens, MDSCs acting as anti-inflammatory tool tries to bring peace between host and pathogen by maintaining immune homeostasis. Cancer and infections are homologous and share common pathophysiology that pushes the hematopoietic system and guides the expansion of MDSCs. Inflammation gets awry and pathogenic during the chronic phase of infection. The progression of disease during chronic infections is directly proportional to the expansion of MDSCs. Recent reports indicated the expansion of MDSCs in various chronic infections caused by bacteria, virus, parasites and fungi. Mostly, M-MDSCs is reported to get expanded in infections caused by Gram-positive bacteria *(M. tuberculosis, Staphylococcus aureus*), Gram negative bacteria (*Klebsiella pneumonia, Helicobacter pylori, Escherichia coli*), polymicrobial sepsis, virus (Hepatitis B virus, HBV soluble antigen, Hepatitis C virus, Human immunodeficiency virus, Influenza A virus, Virus murine acquired immune deficiency syndrome (AIDS), Murine herpesvirus 68) (150). Along with Tregs and IL-10, MDSCs were elevated in viral infection caused due to Japanese encephalitis virus (JEV). JEV induced MDSCs keep the potential to suppress T cells (specifically T[follicular]), splenic and plasma B cells (151) However, particular subset of MDSCs involved in infections caused by Vesicular stomatitis Indiana virus (VSV), adenovirus, Vaccinia, Pulmonary hypertension (PH), Chronic Obstructive Pulmonary Disease (COPD) is yet an enigma (152–154). Zang et al. showed a positive correlation of MDSCs in patients with primary biliary cholangitis (PBC), a type of liver inflammation. They also found high expression of cysteine-rich protein 61 (CCN1) in impaired cholangiocytes and hepatocytes, which regulates expansion and immunosuppressive function of MDSCs (155). Recent studies reported the involvement of G-MDSCs in T cell proliferation and Th2 cytokine production during prosthetic joint infection (PJI). Mechanistically, PJI induced G-MDSCs mainly reduces chronicity of infection by inhibition of pro-inflammatory cytokine production and anti-microbial actions of cytotoxic immune effector cells (156).

Thus, the survival or clearance of invading pathogen depends largely on the nature of pathogen and the duration of infection. It thus decides whether MDSCs reflect their beneficial role, where they show host protective behavior (“Yang”) (157–159) or harmful role, where they increases the disease burden (“Yin”) (160–162).

A better understanding is further needed to explain MDSCs regulation of host pathogen interaction which will give us a clearer picture about their therapeutic application.

### MDSCs in other miscellaneous conditions

Any pathological condition that encompasses inflammatory component provides us a clue and open questions to explore its disturbed immune homeostasis and the potential role of MDSCs. One of the common factors in chronic inflammation associated disorder is immunosuppression. Recent reports present the microenvironment of Alzheimer's patient brain as immunosuppressive, where various chemokines and cytokines secreted by AD brains keep the potential for recruitment and activation of MDSCs which promotes the deposition of AB plaques and is thus involved in its pathology. The exact mechanism of immunosuppression is still not explored much (163). Studies on other neurological disorders like amyotrophic lateral sclerosis (ALS), Parkinson's disorder and Huntington's disease also reports the presence of elevated MDSCs but the exact immune mechanism involved is still a question that needs to be answered (164, 165). Apart from detrimental role of MDSCs (“Yin”) in neurodegenerative diseases, they exhibit cardio protective role (“Yang”) in heart failure patients. Through the secretion of IL-10 and NO, these immune suppressive cells act as anti-hypertrophic and anti-inflammatory molecule. Adoptive transfer of MDSCs alleviates the cardiac dysfunction (166).

## Concluding remarks

The hyper-flexible nature of MDSCs has been enthralling scientific community especially over the last 15 years. Even though we have understood the functionality of MDSCs on an immuno-suppressive and anti-inflammatory level but that development has been in retrospect of cancer studies done over the last decade. We still lag behind in developing methods to modulate the phenotypic responses proactively and we have only been able to define the levels of MDSCs and categorized them.

MDSCs shift their functional phenotypic response as per the given environmental stimuli. On one hand, it enlightens the milieu of pregnancy, where it is crucial during 1st trimester of pregnancy during implantation process; on the other hand it constrains our immune system's response toward developing tumors, and reduces anti-tumor immunity.

With the functional understanding of M-MDSCs and G-MDSCs, we are now able to identify on surface the plasticity of these cells in various types of cancers and in various stages of pregnancy. However, there are still patches of missing information to completely understand the molecular functional mechanisms in differentiation of MDSCs.

The key take home message of this article is to understand how these MDSCs behave as a double edged sword in different pathological conditions. The various diseases and conditions mentioned have been studied from both aspects of beneficial and detrimental effects of MDSCs. Their behavior is critical into unlocking significant clinical and therapeutic progressions in the field of medicine.

With extra-ordinary work going on all around, in few years we would be able to mine the pertinent information to able to identify the markers of these cells and develop precision targeting, opening up interesting areas of research and experimentation in domains for pro-active immune surveillance.

## Author contributions

KS, SB, PV, and SR contributed in the conception and design of the review. SB wrote the first draft of the manuscript. PV contributed in writing the sections of the review. RV helped in critically revising the review for intellectual content.

### Conflict of interest statement

The authors declare that the research was conducted in the absence of any commercial or financial relationships that could be construed as a potential conflict of interest.
